# Expression and regulation of redoxins at nociceptive signaling sites after sciatic nerve injury in mice

**DOI:** 10.1016/j.dib.2015.10.038

**Published:** 2015-11-10

**Authors:** Lucie Valek, Maike Kanngießer, Irmgard Tegeder

**Affiliations:** Institute of Clinical Pharmacology/ZAFES, Goethe-University Hospital, Frankfurt, Germany

**Keywords:** Redoxin, Hypoxia inducible factor 1 alphal, Dorsal root ganglia, Spinal cord, Immunohistochemistry

## Abstract

Injury of the sciatic nerve results in regulations of pro- and anti-oxidative enzymes at sites of nociceptive signaling including the injured nerve, dorsal root ganglia (DRGs), dorsal horn of the spinal cord, thalamus and somatosensory cortex (Valek et al., 2015) [1]. The present DiB paper shows immunohistochemistry of redoxins including peroxiredoxins (Prdx1–6), glutaredoxins (Glrx1, 2, 3, 5), thioredoxins (Txn1, 2) and thioredoxin reductases (Txnrd1, 2) in the DRGs, spinal cord and sciatic nerve and thalamus in naïve mice and 7 days after Spared sciatic Nerve Injury (SNI) in control mice (Hif1α-flfl) and in mice with a specific deletion of hypoxia inducible factor 1 alpha (SNS-HIF1α^−/−^) in DRG neurons. The sciatic nerves were immunostained for the respective redoxins and counterstained with hematoxylin. The redoxin immunoreactivity was quantified with ImageJ. For the DRGs and spinal cord the data show the quantitative assessment of the intensity of redoxin immunoreactivity transformed to rainbow pseudocolors. In addition, some redoxin examples of the ipsi and contralateral dorsal and ventral horns of the lumbar spinal cord and some redoxin examples of the thalamus are presented.

**Specifications Table**

**Table:** 

*Subject area*	*Neuroscience*
*More specific subject area*	*Peripheral nervous system, neurobiology, redox biology*
*Type of data*	*Immunohistochemistry images*
*How data was acquired*	*Immunohistochemistry for redoxins, counterstaining with hematoxylin, microscopy on an Leica Diaplan microscope equipped with a MicroPublisher camera (QImaging, Surrey, BC, Canada)*
*Data format*	*Tables, microscopy images*
*Experimental factors*	*Mice subjected to sciatic nerve injury versus control.*
*Experimental features*	*The data were obtained from mice with a specific deletion of hypoxia inducible factor 1 alpha (SNS-HIF1α*^−/−^*) in sensory neurons of the dorsal root ganglia (DRGs) and floxed control mice.*
*Data source location*	*Frankfurt, Germany*
*Data accessibility*	*Data is with this paper*

## Value of the data

• The immunohistology of redoxins may be used for comparison of the expression and regulation of these enzymes in models of nerve, brain or spinal cord injury.
• The Hif1α dependent regulation of redoxin expression may be used for comparison of redoxin regulation in other tissues e.g. in cancer or cardiovascular tissue.
• The rainbow pseudocolor conversion of quantitative immunhistology data of dorsal root ganglia and spinal cord may be used as an example for quantitative assessment and presentation of immunohistology data.

## 1 Data

**Fig. 1:**
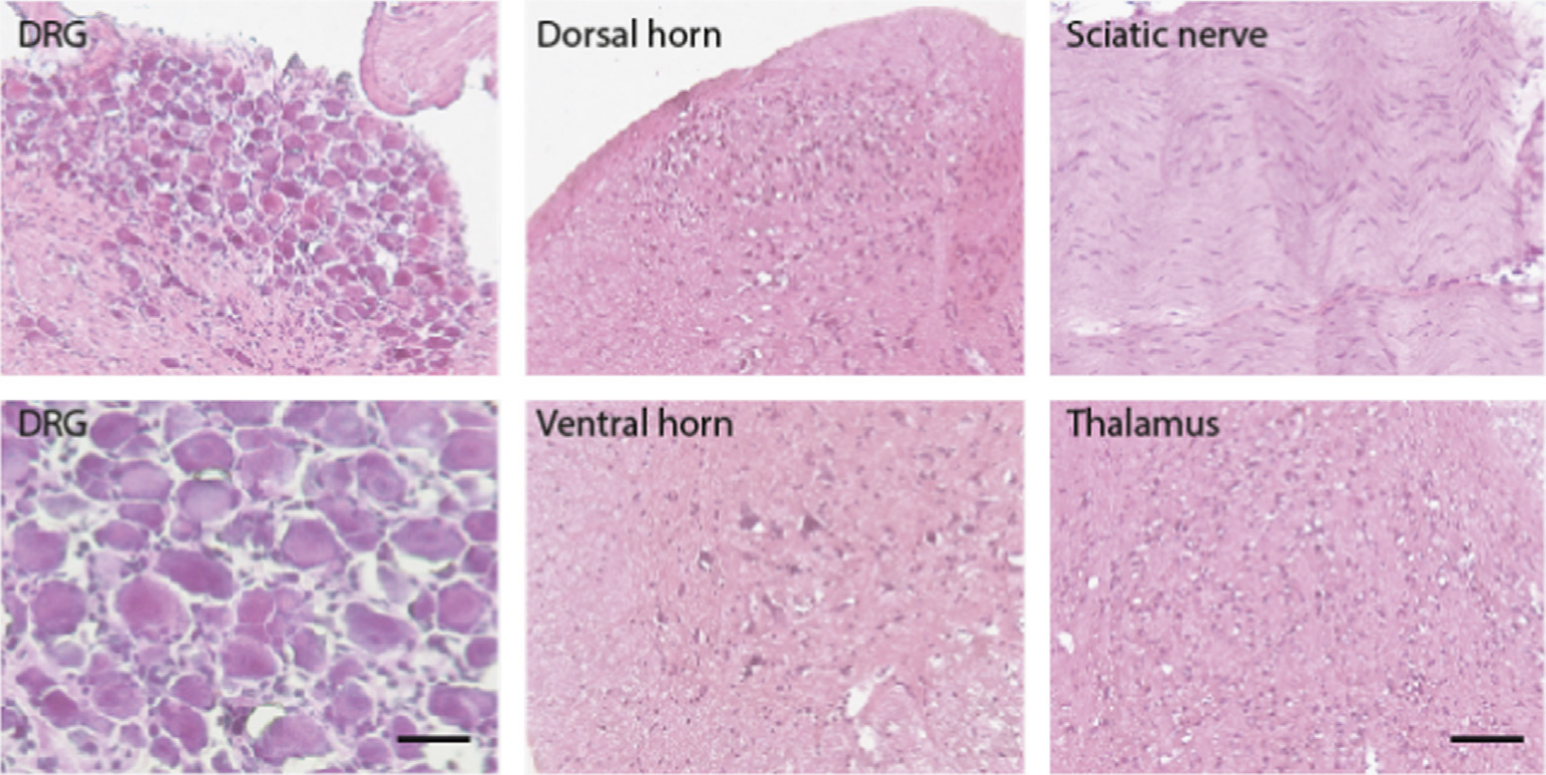
Control stainings of DRGs, dorsal and ventral horns of the spinal cord, sciatic nerve and thalamus. Slides were incubated with anti-Ig *G* without primary antibody and developed with the Streptavidin–HRP system using red AEC as substrate and then counterstained with hematoxylin (blue). Scale bar 20 µm or 50 µm.

**Fig. 2:**
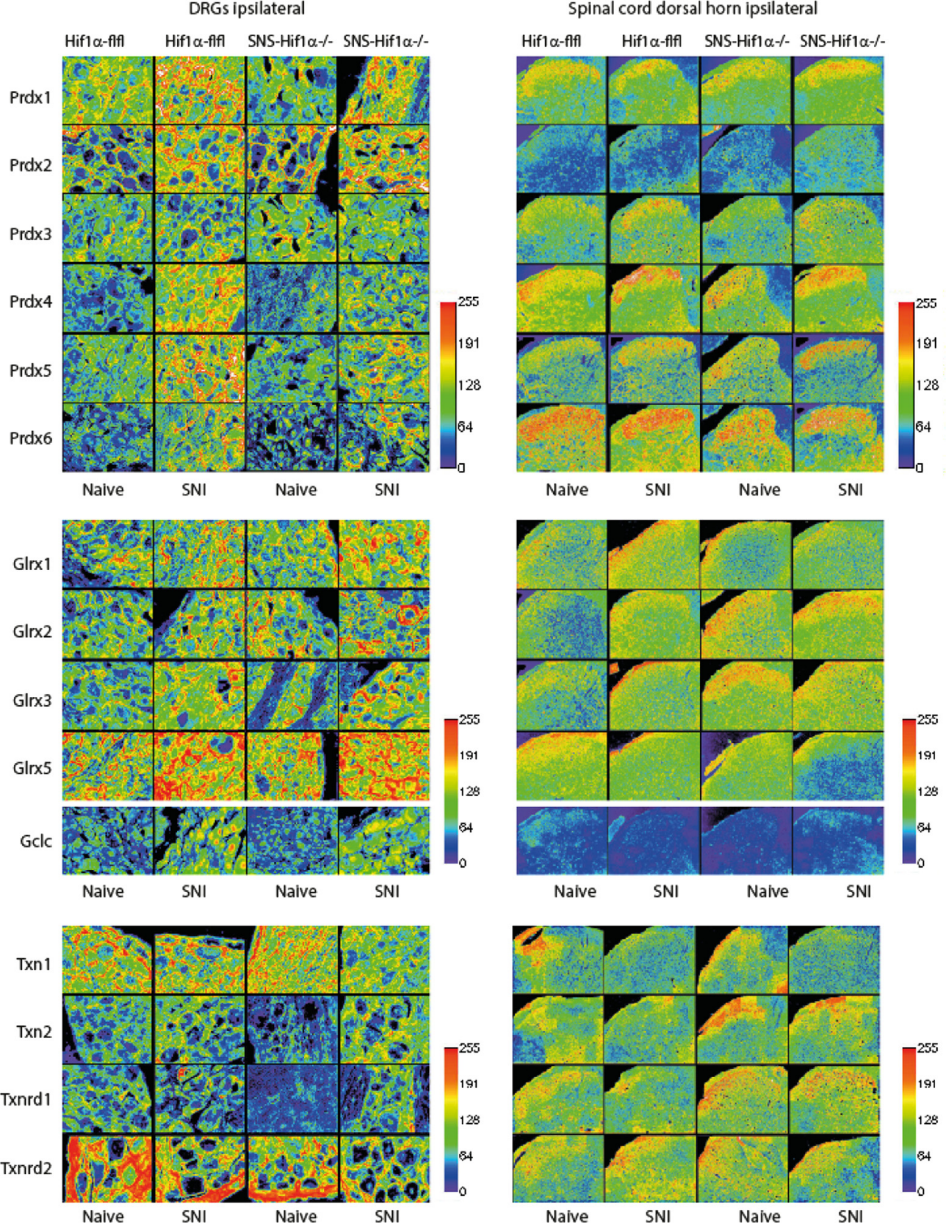
WCIF-ImageJ was used for quantification of histological images. After substraction of background, the RGB image was split into its channels, the channel representing the redoxin-immunoreactivity was then inverted, the threshold set to auto, and intensities transformed to rainbow colors as shown in the images. The mean pixel intensity and distribution were plotted as histograms and the mean intensity was used for statistical comparisons. The quantification is based on results of 3 animals in each group.

**Fig. 3:**
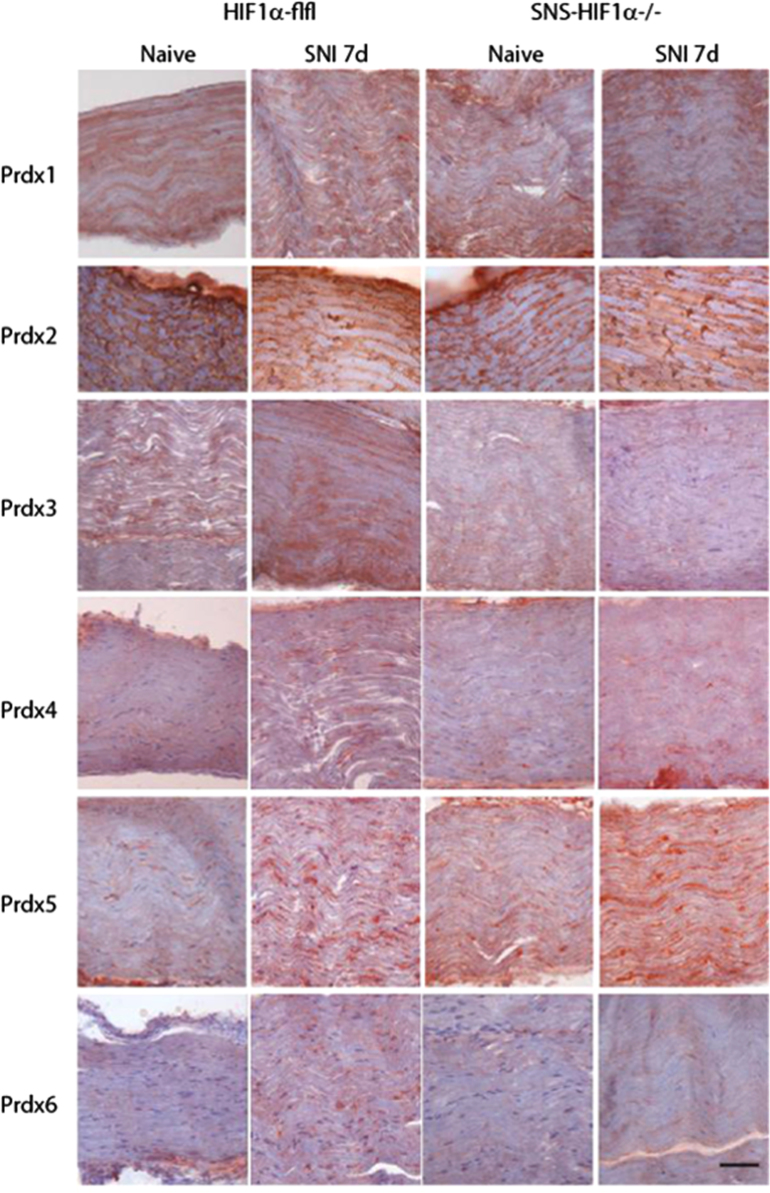
Immunohistology of peroxiredoxins, Prdx1–6 (in red) in the ipsilateral sciatic nerve proximal of the nerve lesion 7 days after Spared Nerve Injury (SNI) in SNS-HIF1α^−/−^ and HIF1α-flfl mice. SNS-HIF1α^−/−^ have a cre/loxP mediated deletion of hypoxia inducible factor 1 alpha specifically in sensory neurons of the dorsal root ganglia (DRGs). Naïve mice were used as controls. Slides were developed with the Streptavidin–HRP system using red AEC as substrate and then counterstained with hematoxylin (blue). Results are representative results of 3 mice per group. Scale bar 50 µm.

**Fig. 4:**
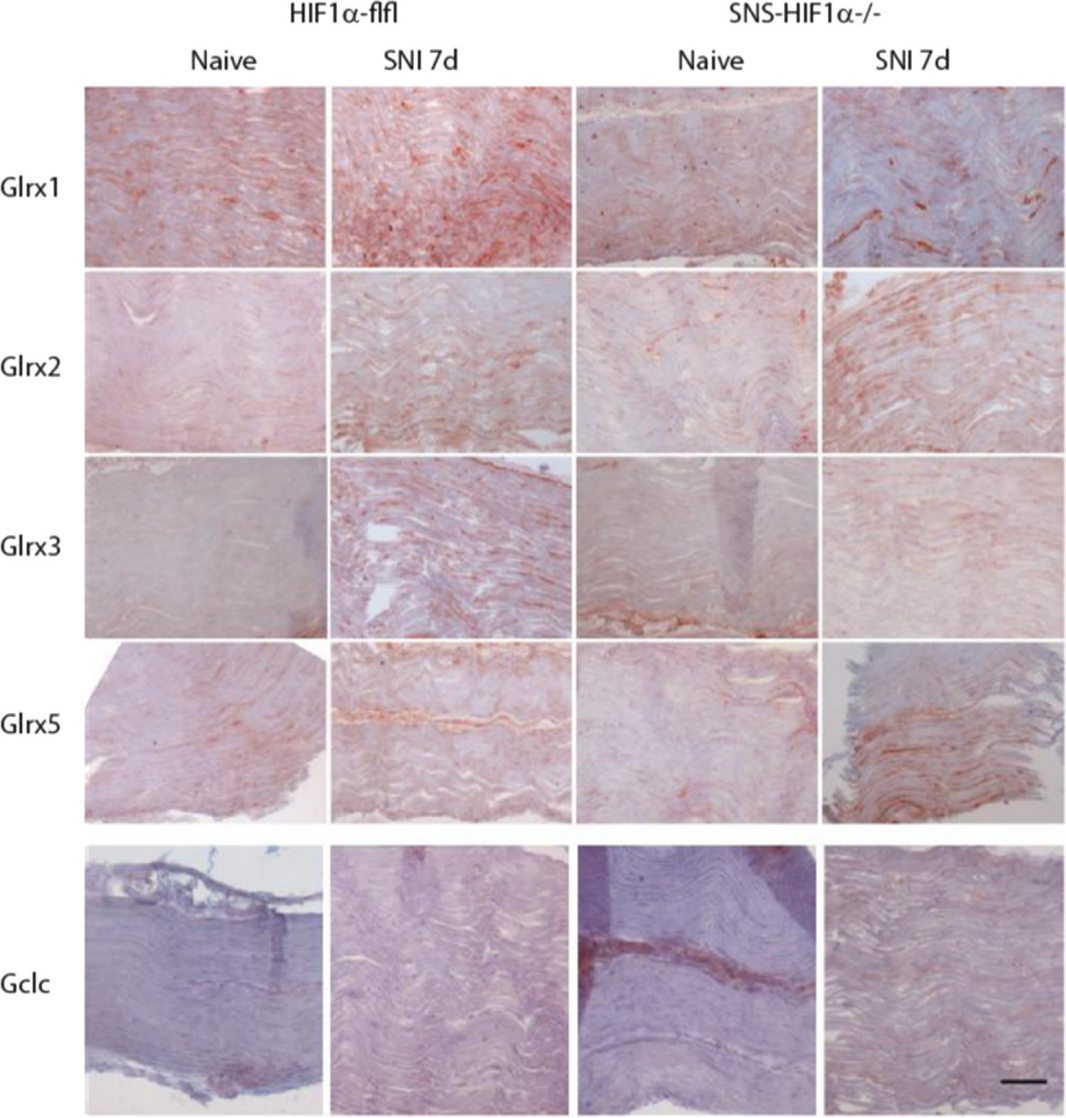
Immunohistology of glutaredoxins, Glrx1, 2, 3 and 5 (in red) and of the catalytic subunit of glutamate–cysteine ligase, Gclc (red) in the ipsilateral sciatic nerve proximal of the nerve lesion 7 days after Spared Nerve Injury (SNI) in SNS-HIF1α^−/−^ and HIF1α-flfl mice. SNS-HIF1α^−/−^ have a cre/loxP mediated deletion of hypoxia inducible factor 1 alpha specifically in sensory neurons of the dorsal root ganglia (DRGs). Naïve mice were used as controls. Slides were developed with the Streptavidin–HRP system using red AEC as substrate and then counterstained with hematoxylin (blue). Results are representative results of 3 mice per group. Scale bar 50 µm.

**Fig. 5:**
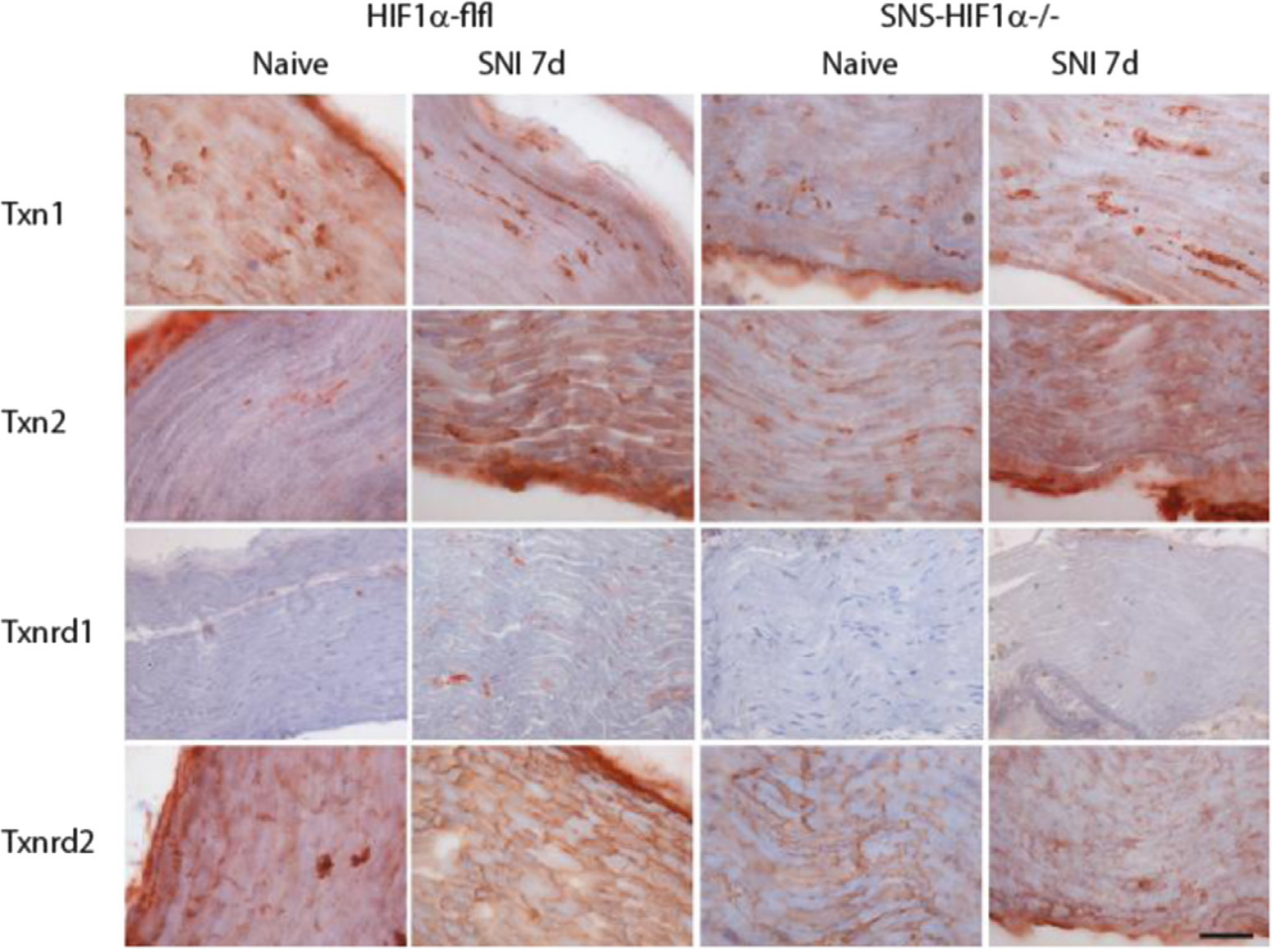
Immunohistology of thioredoxins (Txn1, 2) and thioredoxin reductases (Txnrd1, 2) (in red) in the ipsilateral sciatic nerve proximal of the nerve lesion 7 days after Spared Nerve Injury (SNI) in SNS-HIF1α^−/−^ and HIF1α-flfl mice. SNS-HIF1α^−/−^ have a cre/loxP mediated deletion of hypoxia inducible factor 1 alpha specifically in sensory neurons of the dorsal root ganglia (DRGs). Naïve mice were used as controls. Slides were developed with the Streptavidin–HRP system using red AEC as substrate and then counterstained with hematoxylin (blue). Results are representative results of 3 mice per group. Scale bar 50 µm.

**Fig. 6:**
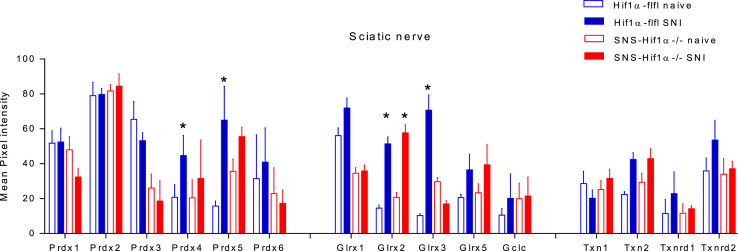
Quantification of redoxin histology of the sciatic nerve with WCIF ImageJ. Mean pixel intensities were determined after substraction of background, RGB split into its channels and threshold settings based on negative control images using “auto” settings. The analysis did not differentiate between different types of cells and is a global readout for immunoreactivities in axonal fibers, Schwann cells and infiltrating immune cells. Pixel intensities were compared with one-way ANOVA for each redoxin separately. In case of significance, groups were mutually compared employing a Sidak correction of alpha, which was set at 0.05 for all comparisons. Asterisks indicate significant differences versus the respective naive animals. The analysis was based on results of 3 animals per group.

**Fig. 7:**
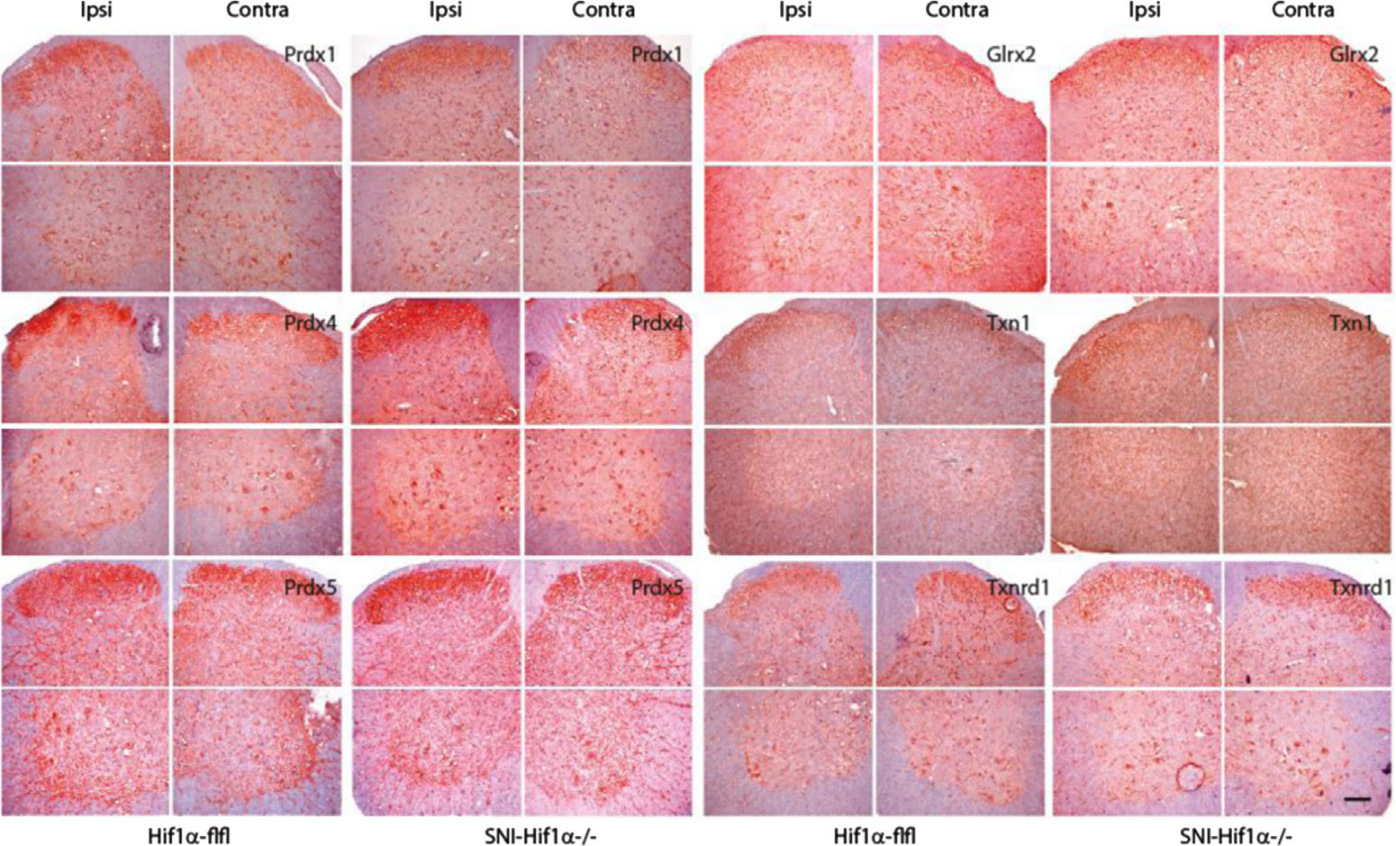
Exemplary immunohistology of redoxins (red, hematoxylin counterstain in blue) in the contralateral and ipsilateral lumbar spinal cord 7 days after Spared Nerve Injury (SNI) of the ipsilateral sciatic nerve in SNS-HIF1α^−/−^ and HIF1α-flfl mice. SNS-HIF1α^−/−^ have a cre/loxP mediated deletion of hypoxia inducible factor 1 alpha specifically in sensory neurons of the dorsal root ganglia (DRGs). Scale bar 50 µm.

**Fig. 8:**
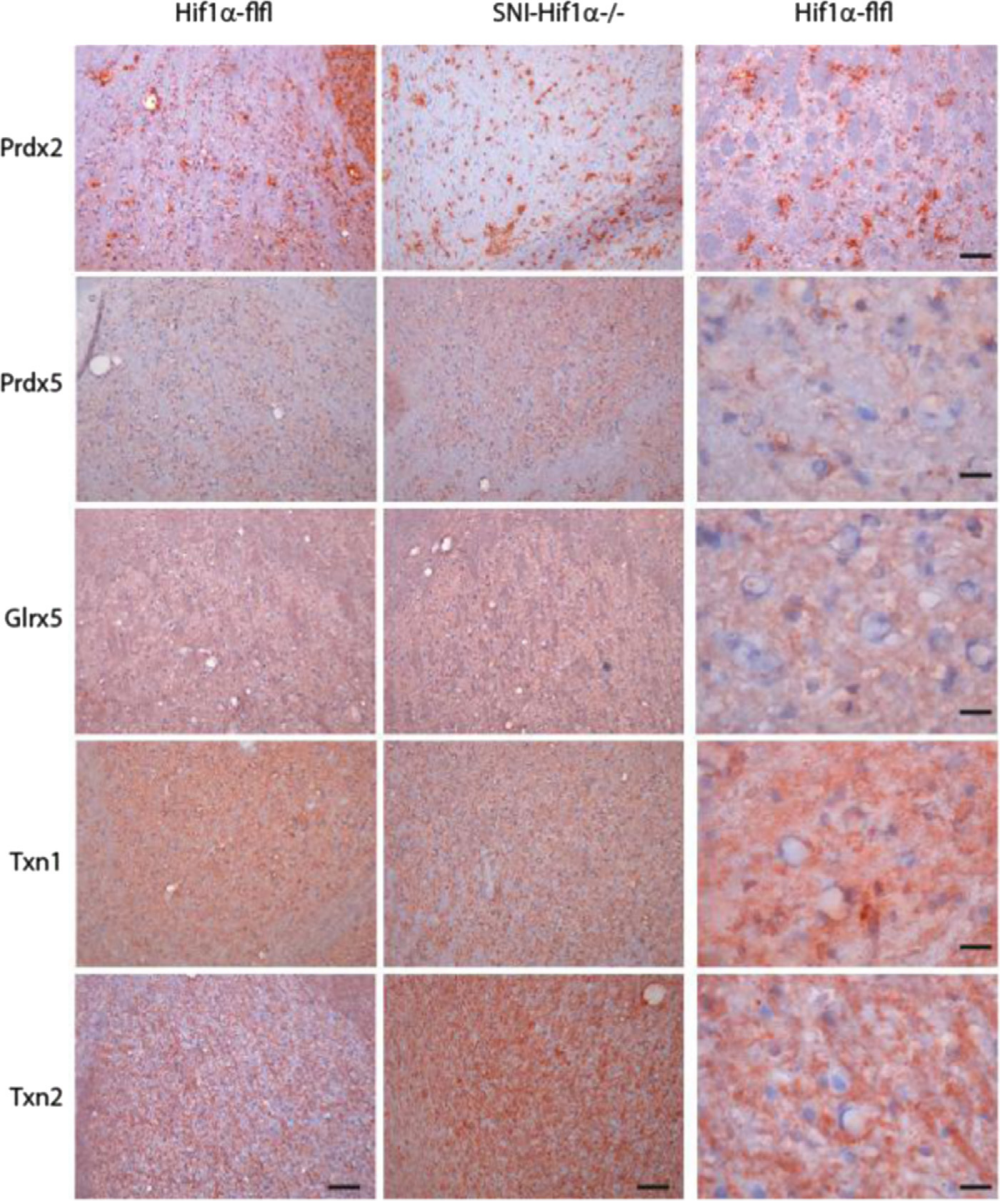
Exemplary immunohistology of redoxins (red, hematoxylin counterstain in blue) in the thalamus 7 days after Spared Nerve Injury (SNI) of the sciatic nerve in SNS-HIF1α^−/−^ and HIF1α-flfl mice. SNS-HIF1α^−/−^ have a cre/loxP mediated deletion of hypoxia inducible factor 1 alpha specifically in sensory neurons of the dorsal root ganglia (DRGs). The right panel shows a higher magnification. Slides were developed with the Streptavidin–HRP system using red AEC as substrate. Scale bars 50 and 20 µm.

The present DiB paper shows immunohistochemistry of redoxins including peroxiredoxins (Prdx1–6), glutaredoxins (Glrx1, 2, 3, 5), thioredoxins (Txn1, 2) and thioredoxin reductases (Txnrd1, 2) in the DRGs Figs. 1 and 2), spinal cord (Fig. 1, Fig. 2, Fig. 7), sciatic nerve (Fig. 3, Fig. 4, Fig. 5, quantification Fig. 6) and thalamus (Fig. 8) in naïve mice and 7 days after Spared sciatic Nerve Injury (SNI) in control mice (Hif1α-flfl) and in mice with a specific deletion of hypoxia inducible factor 1 alpha (SNS-HIF1α^−/−^) in DRG neurons. The sciatic nerves were immunostained for the respective redoxins and counterstained with hematoxylin. The redoxin immunoreactivity was quantified with ImageJ. For the DRGs and spinal cord the data show the quantitative assessment of the intensity of redoxin immunoreactivity [1] transformed to rainbow pseudocolors (Fig. 2). In addition, some redoxin examples of the ipsi and contralateral dorsal and ventral horns of the lumbar spinal cord (Fig. 7) and some redoxin examples of the thalamus (Fig. 8) are presented. Characteristics of the antibodies are listed in Table 1 along with some features of the redoxins.

## 2 Experimental design, materials and methods

### 2.1 Mice and surgery

The data were obtained from mice with a specific deletion of hypoxia inducible factor 1 alpha (SNS-HIF1α^−/−^) in sensory neurons of the dorsal root ganglia (DRGs). The deletion was achieved by mating floxed mice (Hif1α-flfl) with SNScre mice, which express cre-recombinase under control of the Nav1.8/SNS promoter, which is specific for small and medium sized DRG and trigeminal neurons [2]. Hif1α-flfl littermates were used as controls.

Mice were subjected to a sciatic nerve during isoflurane anesthesia using the SNI model [3]. Two of the three peripheral branches of the sciatic nerve, the common peroneal and the tibial nerves, were ligated with silk and distally transected, leaving the sural nerve intact. Seven days after nerve injury mice were terminally anesthetized with isoflurane and cardially perfused with ice cold 1× phosphate buffered saline (PBS), pH 7.4 followed by 4% paraformaldehyde (PFA) in PBS for fixation. Naïve mice were used as controls.

### 2.2 Immunohistochemistry

Tissues were excised, postfixed in 4% PFA for 2 h, cryoprotected overnight in 20% sucrose at 4 °C, embedded in small tissue molds in cryomedium and cut on a cryotome (12 µm for DRGs and sciatic nerves; 18 µm spinal cord). Slides were air-dried and stored at −80 °C. After thawing, slides were immersed and permeabilized in 1× phosphate buffered saline (PBS) with 0.3% Triton-X-100 (PBST), incubated in 3% hydrogen peroxide for 10 min to quench endogenous peroxidase, then blocked with 1% blocking reagent (Roche) or with 10% normal goat serum (Sigma) in PBST, subsequently incubated overnight with the primary antibody in PBST at 4 °C. Primary antibodies are listed in Table 1, validated in [4]. Sections were then washed with PBST, incubated with a biotinylated species specific secondary antibody diluted 1:500 for 1–2 h at room temperature. Vectastain HRP streptavidin was used for antigen detection according to the manufacturer׳s recommendations using red aminoethyl carbazole HRP substrate (AEC, Invitrogen). Subsequently, slides were counter-stained with Mayer׳s hematoxylin and mounted with Mowiol. Negative control slides were incubated with 10% goat serum in PBST, examples of negative control stainings are shown in Fig. 1.

### 2.3 Quantitation of immunohistology images

The WCIF plugin bundle of ImageJ was used for quantification of immunohistology images [5]. After background subtraction the RGB image was split into its channels to separate the immunoreactive red from the blue hematoxylin counterstain. The single channel image was then inverted, the intensity threshold set to automatic detection, the intensity distribution plotted as histogram and the mean intensity was used for statistical comparisons. The intensities were transformed to rainbow pseudocolor for visualization (Fig. 2). The analysis did not differentiate between different types of cells, e.g. neuronal fibers, Schwann cells and immune cells in the nerves. The analysis was based on 2 images per mouse and tissue of 3 mice per group. Mean pixel intensities were submitted to analysis of variance followed by post hoc analysis between treatment groups employing a Sidak correction for multiple testing. *P* was set to 0.05 for all comparisons.

The thioredoxin system consists of the cytosolic Txn1, which can be shuttled to the nucleus and can be secreted [6] or the mitochondrial Txn2 and the respective thioredoxin reductases (Txnrd1 or Txnrd2) [7]. Following disulfide reduction by the respective Txn, Txnrd reduces the disulfide in the active site using electrons provided by NADPH. Glutaredoxins (Glrx) do not depend on a specific reductase for catalysis. Instead, Glrxs use glutathione (GSH) as electron source [8], [9], [10], which is synthesized in two steps by glutamate cysteine ligase (Gclc) and glutathione synthetase [11]. Oxidized GSH, glutathione disulfide (GSSG), is subsequently reduced by glutathione reductase (Gsr) at the expense of NADPH. Peroxiredoxins reduce peroxides rather than protein disulfides [12]. During the peroxidase reaction, the 2Cys Prdxs (1, 2 and 3 and atypical Prdx4 and 5) form a sulfenic acid intermediate, which reacts with a second resolving cysteine, resulting in a disulfide bond, which is subsequently reduced by Txns. The 1Cys Prdx6 uses glutathione (GSH) for the reduction of the sulfenic acid intermediate. Sulfiredoxins prevent the over-oxidation of peroxiredoxins.

## Figures and Tables

**Table 1:** Characteristics of redoxins and antibody specifications.

Name	Gene	GO molecular function	GO cellular component	Type	Catalytic activity	aArnt, Ahr or HIF1 sites	Phosphosites	Antibody
Peroxiredoxin 1	Prdx1	Thioredoxin peroxidase activity, heme binding, antioxidant activity, oxidoreductase activity	N, C, S	2Cys-intermolecular –S–S–	2R′–SH+ROOH=R′–S–S–R׳+H_2_O+ROH	-	Thr90	[4]
Peroxiredoxin 2	Prdx2	Thioredoxin peroxidase activity, antioxidant activity, oxidoreductase activity	C, S	2Cys-intermolecular –S–S–	2R′–SH+ROOH=R′–S–S–R׳+H_2_O+ROH	48	Ser112, Thr182	Santa Cruz Biotechnology, Inc. (sc-33572)
Peroxiredoxin 3	Prdx3	Thioredoxin peroxidase activity, antioxidant activity, oxidoreductase activity	M, C, E	2Cys-intermolecular –S–S–	2R′–SH+ROOH=R′–S–S–R׳+H_2_O+ROH	-	Thr146	[4]
Peroxiredoxin 4	Prdx4	Thioredoxin peroxidase activity, antioxidant activity, oxidoreductase activity	S, C, ER, M, L	atypical 2Cys, intramolecular–S–S–	2R′–SH+ROOH=R′–S–S–R′+H_2_O+ROH	121, 211	Ser68, Tyr266	Abcam plc (ab59542)
Peroxiredoxin 5	Prdx5	Thioredoxin peroxidase activity, antioxidant activity, oxidoreductase activity, RNA polymerase and receptor binding	C, M, N, P	atypical 2Cys, intramolecular–S–S–	2R′–SH+ROOH=R′–S–S–R׳+H_2_O+ROH	125, 404, 221	Ser34, Thr97, Ser101, Ser182	[4]
Peroxiredoxin 6	Prdx6	Glutathione peroxidase activity, phospholipase A2 activity, antioxidant activity, oxidoreductase activity	C, L, S	1Cys	2R′–SH+ROOH=R′–S–S–R׳+H_2_O+ROH; 2 glutathione+H2O2=glutathione disulfide+2 H2O.	102	Ser32, Thr44, Ser83, Tyr89, Ser146, Thr177, Ser186	Abcam plc (ab59543)
Glutaredoxin 1	Glrx1	Glutathione disulfide oxidoreductase activity	C, N, M, S	dithiol	2 Glutathione+H2O2=glutathione disulfide+2 H2O, in the presence of NADPH and Gsr	-	Tyr25	[4]
Glutaredoxin 2	Glrx2	Glutathione disulfide oxidoreductase activity, protein disulfide isomerase activity	M, N	dithiol	2 Glutathione+H2O2=glutathione disulfide+2 H2O, in the presence of NADPH and Gsr	-	Ser20, Tyr103, Tyr113	[4]
Glutaredoxin 3	Glrx3	Protein disulfide oxidoreductase activity, protein disulfide isomerase activity, protein kinase C binding	C, N	monothiol 2×	2 Glutathione+H2O2=glutathione disulfide+2 H2O, in the presence of NADPH and Gsr	34	Ser32, Ser117, Ser120, Ser196	[4]
Glutaredoxin 5	Glrx5	Protein disulfide oxidoreductase activity, 2 iron, 2 sulfur cluster binding, metal ion binding	M, N, C	monothiol	2 Glutathione+H2O2=glutathione disulfide+2 H2O, in the presence of NADPH and Gsr	-	Ser41, Ser145	[4]
Glutamate cysteine ligase, catalytic domain	Gclc	Glutamate–cysteine ligase activity, glutathione synthase activity, ATP binding	C		ATP+l-glutamate+>l-cysteine=ADP+phosphate+gamma->L-glutamyl-l-cysteine	317, 328	Ser5, Ser8, Ser621	Santa Cruz Biotechnology, Inc. (sc-22755)
Thioredoxin 1	Txn1	Protein disulfide oxidoreductase activity, poly(A) RNA binding, dithiol–disulfide exchange	C, N, S	cytopslasmic	Dithiol–disulfide exchange	-	Ser44, Ser46, Ser67	[4]
Thioredoxin 2	Txn2	Protein disulfide oxidoreductase activity, dithiol–disulfide exchange	M, C, N	mitochondrial	Dithiol–disulfide exchange	155 (mouse)	-	[4]
Thioredoxin reductase 1	Txnrd1	Selenium-dependent oxidoreductase activity, thioredoxin–disulfide reductase activity, NADP(H) oxidase activity	C, N	cytopslasmic	Thioredoxin+NADP^+^=thioredoxin disulfide+NADPH.	461	Tyr161, Tyr163, Tyr277, Tyr281	[4]
Thioredoxin reductase 2	Txnrd2	Selenium-dependent oxidoreductase activity, thioredoxin–disulfide reductase activity, acting on a sulfur group of donors, NAD(P) as acceptor	M	mitochondrial	Thioredoxin+NADP^+^=thioredoxin disulfide+NADPH.	118	Tyr40	Santa Cruz Biotechnology, Inc. (sc-67127)

M, Mitochondria; C, Cytoplasm; N, Nucleus; V, Vesicular structure (endosome, peroxisome, lysosome); E, Endosome; P, Peroxisome; S, Secreted; ER, Endoplasmic reticulum; L, Lysosome.

a Search 500 kB upstream.
